# Is the VO_2_max that we measure really maximal?

**DOI:** 10.3389/fphys.2013.00203

**Published:** 2013-08-05

**Authors:** Bruno P. C. Smirmaul, Danilo R. Bertucci, Inaian P. Teixeira

**Affiliations:** ^1^Department of Physical Education, São Paulo State University (UNESP)Rio Claro, Brazil; ^2^Department of Physiological Sciences, Federal University of São Carlos (UFSCAR)São Carlos, Brazil

## Introduction

The maximal oxygen uptake (VO_2_max) can be defined as the maximum integrated capacity of the pulmonary, cardiovascular and muscular systems to uptake, transport and utilize O_2_, respectively (Poole et al., 2008). Usually measured by the incremental exercise test in the treadmill or cycle ergometer, the VO_2_max test has become a cornerstone in clinical and applied physiology involving physical exercise. Its applications are numerous, ranging from elite athletes to individuals with several pathologic conditions (Mancini et al., 1991; Bassett and Howley, 2000). Despite studied for approximately a century, questions regarding the VO_2_max are still source of debate and disagreement in the literature (Noakes, 1998; Bergh et al., 2000; Levine, 2008; Ekblom, 2009; Noakes and Marino, 2009; Spurway et al., 2012). In particular, the study of the methods of VO_2_max measurement is a field of investigation that has been challenging through the years (Midgley et al., 2007, 2008). Intriguing findings recently published (Beltrami et al., 2012; Mauger and Sculthorpe, 2012) bring additional debate regarding the measurement of the *true* VO_2_max value and its limiting/regulatory mechanisms. In this article we briefly describe the current testing methods and mechanisms of VO_2_max limitation/regulation, and discuss the new findings of these two recent studies and their possible implications in the field.

## Current measurement and VO_2_max limiting/regulatory mechanisms

One of the most popular concepts used to obtain VO_2_max during an incremental exercise test is the occurrence of the plateau. The origin of this concept had its basis in the studies of Hill and Lupton (1923) 90 years ago, in which they proposed the existence of an individual exercise intensity beyond which there is no increase in the VO_2_, representing the limit of the cardiorespiratory capacity. However, the need for the plateau occurrence to the VO_2_max determination presents limitations, once it conflicts with the fact that its occurrence is not universal (Doherty et al., 2003; Astorino et al., 2005). With the purpose to solve this problem and ensure that individuals attain always “maximal” conditions by the end of an incremental exercise test, producing *true* VO_2_max values, the use of physiological parameters as criteria for exercise test interruption based upon respiratory exchange ratio, maximal heart rate and blood lactate concentrations became popular (Poole et al., 2008). These parameters, though, when used as criteria for VO_2_max determination, can underestimate the actual measured value up to 26% (Poole et al., 2008). Finally, the current solution proposed to VO_2_max determination when the plateau does not occur, is the use of the VO_2_ peak, which seems to be a consistent VO_2_max index, as long as a constant supramaximal exercise test is done after the incremental test, called “verification phase” (Day et al., 2003; Midgley and Carroll, 2009).

Presently, two main theoretical models are discussed in the literature aiming to explain the mechanisms of VO_2_max limitation and/or regulation. The classical model proposes that VO_2_max is limited by the maximal capacity of the heart to provide O_2_ to the muscles, that means, when one reaches the VO_2_max the cardiovascular system is working on its limit (Ekblom, 2009). Alternatively, the other model advocates that the cardiovascular system never reaches a limit of work, and that VO_2_max is regulated, rather than limited, by the number of motor unit recruited in the exercising limbs, which is always submaximal (Noakes and Marino, 2009). Thus, this model proposes that there is always a physiological reserve, both cardiovascular and neuromuscular, once the number of motor unit recruited by the active muscles during exercise is regulated by the brain to prevent catastrophic failure in bodily systems (Noakes and Marino, 2009).

## Is the VO_2_max that we measure really maximal?

Independently of the VO_2_max limiting/regulatory mechanisms (Ekblom, 2009; Noakes and Marino, 2009), it is believed that implementing specific criteria during the incremental exercise test as duration (Midgley et al., 2008), presence of the “verification phase” (Day et al., 2003; Midgley and Carroll, 2009), and rate of VO_2_ sample acquisition (Astorino, 2009), one obtains *true* VO_2_max values. Two recent studies, however, challenge such beliefs.

**Figure 1:**
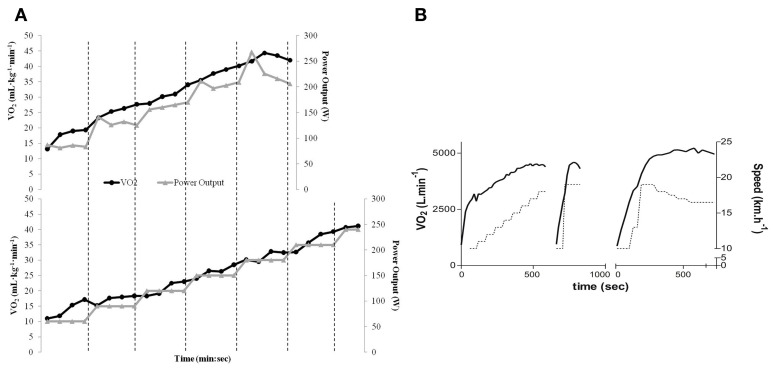
**(A)** VO_2_ and power output data for the self-paced incremental protocol (top) and conventional incremental protocol (bottom) in a representative subject. A higher VO_2_max (group mean ≈8%) was achieved in the self-paced incremental protocol during submaximal workload. **(B)** VO_2_ and speed data for the conventional incremental test (left) + verification phase (middle) and for the decremental protocol (right) in a representative subject. A higher VO_2_max (group mean ≈4.4%) was achieved in the decremental protocol during submaximal workload. VO_2_ is represented by solid lines, and dotted lines represent speed. “Reproduced from Mauger and Sculthorpe (2012) and Beltrami et al. (2012) with permission from BMJ Publishing Group Ltd.”

The first study (Mauger and Sculthorpe, 2012) compared a conventional incremental exercise test (i.e., with fixed load increments until voluntary exhaustion) with a maximal self-paced incremental exercise test regulated by individual perception of effort. The total duration of the latter was 10 min, distributed in 5 stages of 2 min each, in which individuals controlled the exercise intensity at each moment in order to achieve individual perceptions of effort of 11, 13, 15, 17, and 20, respectively, in the 15-points Borg scale. Interestingly, this maximal self-paced incremental test resulted in a significantly higher VO_2_max (≈8%; Figure 1A) when compared to the values found during the conventional incremental exercise test (Mauger and Sculthorpe, 2012).

The second study (Beltrami et al., 2012) compared a conventional incremental exercise test with a decremental protocol (i.e., with decreasing exercise intensity levels over time). This decremental protocol started in the speed used during the “verification phase” of the incremental test, which means, 1 km h^−1^ faster than the last stage accomplished during the conventional exercise test. This intensity was kept for 60% of the individual time that subjects were able to tolerate during the “verification phase,” with a subsequent reduction in speed of 1 km h^−1^ for 30 s and consecutive reductions of 0.5 km h^−1^, in which each stage was kept for 30, 45, 60, 90, and 120 s, respectively. Similarly to the maximal self-paced incremental test (Mauger and Sculthorpe, 2012), the decremental test proposed resulted in significantly higher VO_2_max (≈4.4%; Figure 1B) when compared to the conventional incremental exercise test (Beltrami et al., 2012).

The main explanation suggested by the authors for the results found in the first study (Mauger and Sculthorpe, 2012) is that the nature of the self-paced protocol may have allowed a higher power output for the same level of perception of effort or discomfort, leading to greater VO_2_max before voluntary exhaustion. This occurred despite heart rate, ventilation, and respiratory exchange ratio values being similar to the conventional protocol. Additional suggestions as a greater relative contribution of oxygen-dependent type 1 fibers with a consequent reduction in the anaerobic component of the test, and/or an increase in the oxygen demand and utilization due to the high power output in the last stage of the self-paced incremental test, may also have contributed to the greater VO_2_max found (Mauger and Sculthorpe, 2012). It is noteworthy that criticisms have already been raised to this study (Chidnok et al., 2013). At the same time the authors of the second study (Beltrami et al., 2012) suggest that differences in the anticipatory workload perception of the protocols, growing in the conventional incremental test and reducing in the decremental test, might have impacted the sympathetic or parasympathetic drives and led to different metabolic responses to exercise and to the greater VO_2_max. Surprisingly, both studies showed that either untrained (Mauger and Sculthorpe, 2012), or trained (Beltrami et al., 2012) individuals attained the greater VO_2_max values during submaximal workloads, challenging the traditional concept that VO_2_max occurs at the maximal workload.

## Implications of the new findings

Once recognized and further corroborated that current VO_2_max measurement methods (i.e., conventional incremental exercise protocol) provide, in fact, submaximal values, which would be the implications of the new *true* VO_2_max values found (Beltrami et al., 2012; Mauger and Sculthorpe, 2012) upon the existing body of knowledge relating to this area? In our opinion, a considerable portion of the scientific knowledge would be mildly affected, due to the existence of systematic error. For instance, studies aiming to verify the effect of specific interventions upon VO_2_max already have VO_2_max underestimations aggregated into their results. As pre- and post- intervention values are measured by the same protocol, the intervention effects upon VO_2_max values would still be correctly measured, despite underestimation of VO_2_max *true* value. In contrast, studies based upon VO_2_max percentages, as the aerobic training zone for cardiorespiratory fitness, for example, which habitually varies around 50 and 85% of VO_2_max, would have its interval range shifted to the right. Likewise, it would be necessary to review the indirect equations to estimate VO_2_max, as they make use of VO_2_max reference values that are, according to the new findings (Beltrami et al., 2012; Mauger and Sculthorpe, 2012), submaximal. Nevertheless, knowing the underestimation magnitude of the VO_2_max by the conventional incremental protocols, mathematical equations would be able to provide a posteriori corrections, reducing/correcting such inaccuracies.

Contrary to the relatively minor impact described above, the findings of greater VO_2_max than the ones commonly found during conventional incremental exercise tests conflict with the theoretical models proposed to explain its limiting/regulatory mechanisms (Ekblom, 2009; Noakes and Marino, 2009). If the VO_2_max values found so far during conventional incremental tests are limited by the maximal capacity of the heart to provide O_2_ to the muscles (Ekblom, 2009), how can one explain such an increase (Beltrami et al., 2012; Mauger and Sculthorpe, 2012)? We identify two possibilities. The theoretical model may still be correct, that means, VO_2_max is indeed limited by the maximal capacity of the heart, though, the VO_2_max values found during conventional incremental tests are not *truly* maximal, and alternative protocols would be able to increase it. In opposition, the model may be wrong in stating that VO_2_max is primarily limited by the cardiac capacity, and another mechanism might exist to explain its limitation/regulation. The other theoretical model (Noakes and Marino, 2009), on its turn, also conflicts with the findings. If the brain regulates the number of motor unit recruited during exercise in order to prevent catastrophic failure in bodily systems, thus regulating the VO_2_max achievable, why would the brain allow individuals during these two new protocols (Beltrami et al., 2012; Mauger and Sculthorpe, 2012) to attain VO_2_max values greater than during the conventional incremental tests? Would not the brain, based on afferent feedback from various systems, regulate the number of motor unit recruited in a similar fashion, independently of the exercise protocol performed?

A possible explanation for the recent findings may be found reaching back to the proposal by Jones and Killian (2000), who reviewed evidence to show that, rather than limitations based on the capacity of oxygen-delivery mechanisms, cardiorespiratory and exercise limitations are symptom-based. These authors, considering peripheral and central perceptions of effort data, raised the importance of considering these symptoms as limiting factors when measuring exercise performance and VO_2_max (Jones and Killian, 2000). A recent theoretical model further emphasizes the paramount importance of effort on endurance exercise performance regulation and tolerance (Marcora and Staiano, 2010; Smirmaul et al., 2013). The higher VO_2_max values achieved (Beltrami et al., 2012; Mauger and Sculthorpe, 2012) may have been associated with altered perceptual responses due to the differences in the protocols used. However, this possibility remains speculative.

## Conclusion

The proposals of different exercise protocols which result in greater VO_2_max values than commonly found during the conventional incremental exercise tests should interest the exercise and sports physiology community. At the same time that such findings mildly impact a considerable portion of knowledge, they challenge, for instance, the theoretical models to explain VO_2_max limitation/regulation. Still, they also challenge the concept that VO_2_max occurs at the maximal workload. While recent work has shown that it is possible to maintain a conventional VO_2_max plateau up to 15 min by decreasing individuals' workload, that means, during submaximal work (Petot et al., 2012; Billat et al., 2013), it is unknown whether the same is possible for the superior VO_2_max values found (Beltrami et al., 2012; Mauger and Sculthorpe, 2012). The suggestion that VO_2_max values are task-dependent, and that the conventional incremental exercise test does not produce *true* maximal values is attractive. However, understanding how these new exercise protocols produce higher VO_2_max values, the influences of different protocols on perceptual responses and VO_2_max measurement, determining its full implications and applications, and the specific limiting/regulatory mechanisms underpinning VO_2_max, are new horizons that sports and exercise scientists may explore.
